# The Queen's Jubilee Hospital

**Published:** 1905-04-22

**Authors:** Frederick M. Fry, J. Danvers Power


### THE QUEEN'S JUBILEE HOSPITAL.

The following report embodies the result of an inquiry into the affairs of this hospital by King Edward's Hospital Fund for London.

### Introductory.

Letters from the Chairman of the Queen's Jubilee Hospital, requesting the Fund to inquire into the recent trouble at the hospital and into the question of its general management, were considered at a meeting of the authorities of King Edward's Hospital Fund held on March 30, 1905; and it was resolved "that the executive committee undertake the inquiry invited by the Queen's Jubilee Hospital, and request Sir Thomas Smith and Mr. H. C. Smith, the visitors, and Mr. Frederick M. Fry and Mr. J. Danvers Power, to make a report to them on the subject, after such investigations on the spot as they may think desirable."

Sir Thomas Smith being unable to attend and Mr. H. C. Smith being abroad, the following report was made by Mr. Frederick M. Fry and Mr. J. Danvers Power.

### Report and Recommendations.

We have held three long sittings at the hospital, namely, on April 6th, 7th, and 8th, and we paid a visit of inspection on April 10th. The Board of Management were represented by Mr. Tom Green and Mr. Bavin, and the late medical staff by Mr. R. P. Brooks, F.R.C.S. From these gentlemen we received every assistance. They called before us 23 witnesses, and addressed us on the various questions at issue.

We beg to report as follows:—

1. The Queen's Jubilee Hospital, which stands in the Richmond Road on the east side of the Brompton Cemetery, has 15 beds and two cots, and was founded in 1887 by Mr. Robert Fitzroy Benham, M.R.C.S., a general practitioner living in Earl's Court Square, S.W.

2. Mr. Benham informed us, and the statement was not disputed, that he has himself contributed £3,852 to the general funds of the hospital.

3. Under these circumstances it would seem to be only just and reasonable that the Founder should be elected to a seat on the Board of Management. But, if he wished to set the hospital on a level with other London hospitals, the question of his acting as one of the honorary medical staff should, at the least, have been settled on its merits, and without reference to the acts of apparent munificence to which we have referred. Unfortunately this does not appear to have been the case.

4. We believe it to be generally accepted that the honorary surgeons of recognised London hospitals, should, at the least, be Fellows of a Royal College of Surgeons, and indeed Rule 14 of the revised constitution of the Queen's Jubilee Hospital provides that "all surgeons who shall in future be appointed shall be Fellows of the Royal College of Surgeons, England, Edinburgh, or Ireland.

5. Mr. Benham would not be qualified under this rule, but as he was an existing member of the staff at the time it was passed, he stands outside it. If, therefore, the rule was made in the interests of the hospital it may be assumed that Mr. Benham continues to act on the staff for special reasons personal to himself. Unless these are connected with his recognised professional eminence, as distinct from academic qualification, the only conclusion we can draw is that he occupies his position as a member of the medical staff in virtue of being the founder of the hospital. Should this be the case his position is a false one.

6. The hospital is governed by a Board of Management, which consisted, in 1904, of 28 lay members. Of this number we ascertained that at least nine were not qualified under the rules to serve on the Board. In addition to the lay members all the members of the honorary medical staff are members of the Board.

7. The late medical staff, with the exception of Mr. Benham and the dental surgeon, resigned on February 8th, 1905. They ceased to perform their duties on or about March 8th, when the new staff took over the work.

8. We are not called upon to express any opinion, from a professional point of view, as to the proper course to be pursued by medical men who are invited to form the staff of a hospital immediately after the resignation in a body of any considerable portion of the late staff. Individually and collectively the new staff may have acted quite correctly, for anything we can say to the contrary.

9. But, in the interests of good order, we cannot doubt that, after an event of such importance as the resignation of the medical staff of a hospital, the Board of Management should call for an immediate and independent inquiry; and that such inquiry should be held before, and not after, the election of successors to those who have resigned. Thus that the final settlement would not be unnecessarily complicated by the existence of a third body.

10. In the case in question there is no reason whatever to suppose that, had such an independent inquiry been suggested, the late medical staff would not have been willing to carry on the work until the result was known. Indeed they offered to, and did, carry on the work until their successors were appointed. It is, however, only fair to the Board to state that the late medical staff did not themselves suggest an inquiry, and that they intended and expected their resignations to be accepted.

11. In regard to the management of the hospital prior to the resignation of the late staff, we have no doubt that it was slack and unsatisfactory. It is perhaps not to be expected that the *ex-officio* professional members of the Board should be held equally responsible with the lay members for the details of business management. At the same time we think they would have acted more wisely had they taken steps to make it clearer on the face of the minutes how and when they actively differed from their lay colleagues, and we think the actual occasion of their resignation was not the best which might have been chosen. In saying this we do not, after inquiry into the circumstances, think Dr. Morrison was in any way to blame for the letter to which the Board took exception.

12. We hesitate to use strong terms in regard to voluntary work kindly undertaken by men, many of whom were closely engaged during the day in their own business as tradesmen, and who, after all, had no object to serve except the cause of charity. At the same time we cannot overlook the description of their work given to us by one of their own number. This gentleman, Mr. Tom Green, who at the first sitting was himself the chosen spokesman of the Board of Management, stated that he had been struck with the want of method and unpunctuality of the Board, with the fact that the committees did not regularly report to the superior body, and with "the general atmosphere of latent hostility and suspicion" between the various sub-committees. He informed us that he had never read the rules, that he was unable to account for his having thought that he was qualified to serve on the Board when such, in fact, was not the case, and that he had never heard of an outbreak of diphtheria which took place recently in the nurses' home, until the incident was mentioned before us at the inquiry.

13. This view was partly corroborated by another member of the Board, and the general evidence adduced by the late staff leads us to believe that it is not an unfair statement of the facts. Another member of the Board affirmed that he did not consider it to be the duty of the Board to take cognisance of want of cleanliness in the hospital.

14. It is clear to us that the work of the hospital was to a great extent delegated to committees whose work was not properly brought before the Board. The functions of the Board were indefinite; its control was not always exercised when it should have been, and it sometimes made itself felt in an improper manner. Such, for instance, were the cases alluded to in the first reason given by the late medical staff for their resignations. The salaries offered were insufficient to attract nurses of the class required. We may mention that one of the witnesses called by the Board of Management had been herself engaged as a staff nurse at a salary of £20 a year; in the whole course of her training she had never remained as long as seven months in any one hospital. In these circumstances she was engaged by the matron as staff nurse on presenting her certificates, and without any direct communication with her previous employers. In another case the Board of Management refused to dismiss a nurse whose work was reported to be unsatisfactory, and the reason given to us by one of their number was that she was related to a member of the Board, who it was thought, might influence subscriptions to the hospital. The late matron explained to us that the insubordination among the nurses was due to their knowledge that the Board had refused to dismiss two nurses whose conduct had been complained of, and that her reason for not dealing with a subsequent case, referred to in the statement by the Board, was that she had determined to resign. The following are the terms of this matron's resignation:—

> "Having given the matter my most earnest consideration, and acting on the advice of my father and best friends, I beg to tender to you my resignation. I find it impossible to any longer act as matron to this Institution. I may say that my chief reason for resigning is the constant opposition and interference that I have met with in the proper carrying out of my duties.
>
> "It can, perhaps, do no good to dwell upon the fact, but nevertheless no matron, in my opinion, will ever stay while the Institution is worked on its present lines."

15. A previous matron reported to us that she found the hospital "in a state of filth." The present temporary matron, who acted in a like capacity for two months in 1904, stated that she found the hospital in a "dirty condition" on the first occasion, and in a "worse condition" when she entered upon her present duties.

16. We should add in reference to the member of the medical staff who was said to have relegated his duties to a substitute, that the circumstances were at the time reported to and approved by the medical committee, who, as stated by the Board, "had it in their power to regulate the working of their department according to their own views," and that they were not objected to at the time by the Board. The Board, indeed, appear to have been unaware of the facts, which seem to have been elicited subsequently as the result of inquiries made for the purpose of establishing a case to lay before the King's Fund. The same remark applies to the complaint of the late attendances in the out-patient department made against another member of the staff. The charge against the ophthalmic surgeon we dismiss as unfounded. No corroborated evidence in support of the charges against the resident medical officer was brought before us. The constant changes in the nursing staff under the late matron were accounted for to our satisfaction; but we are not called upon to express any opinion on her general government of the nursing department, except in so far as it came within the duties of the Board and the staff to deal with the matter, and we do not do so.

17. The reference we have already made to Dr. Morrison's letter, set forth in the Board's statement, is the only one we propose to make in connection with the incident of the proposed opening of the new building. The question for us is whether the hospital has been, and is being, properly conducted. That it has not been well conducted we have no doubt. We were informed by a member of the new staff, that the hospital is now in good working order, and that the Board has given the new staff a free hand. We do not consider that the old staff had a free hand, and this fact emphasises our regret that the new staff was elected before any inquiry had been held, especially as the new staff has been increased in size. It now consists of two physicians, two surgeons, one assistant surgeon, one physician for diseases of women, an ophthalmic surgeon, an assistant ophthalmic surgeon, a nose and throat surgeon, an aural surgeon, a physician and an assistant physician for diseases of the skin, besides three anæsthetists, a dentist, a resident house physician and a resident house surgeon; or 18 members, a number in excess of the total number of beds and cots in the hospital.

18. We found the hospital clean, and, as far as its age and capacity allows, in a satisfactory condition. But the conclusion we have come to is that the wards are not suited for the purposes of a general hospital for in-patients.

19. We do not think that even a building on a large scale for in-patients is required on this spot, having regard to the pressing needs of other parts of London. We think, however, that as a casualty and out-patient department with a few beds attached, a properly constructed building might do useful work in the neighbourhood. However this may be, we are satisfied that the extension of ward accommodation is at present unnecessary, and that drastic changes in the management are required.

Recommendations.

20. We are glad to know that such of our recommendations as may be endorsed by the Executive Committee of the King's Fund are likely to be adopted, and it was with satisfaction that we heard the following words from Mr. Bavin:—

"I am perfectly satisfied, on behalf of the Board of Management, to leave the matter in the hands of the commissioners, knowing well that a fair and correct conclusion will be arrived at by them, and hoping that the hospital may arise out of its troubles, and be established on a firmer footing and on a stronger basis than we ever, in our most sanguine moments, hoped for."

21. The advice we offer is that a period of, if possible, 12 months be placed between the present and the future permanent management of the hospital, and that all the members of the present staff and Board should resign, the treasurer and trustees continuing to perform their present duties.

22. We further recommend the governors to agree to a suspension of the constitution of the hospital and the rules and bye-laws for a period of 12 months, and to elect, on the nomination of the executive committee of the King's Fund, a few gentlemen as a Board of Management to carry on the affairs of the hospital for that period under such temporary rules as they may find it desirable to frame. A new Board of Management, so constituted, should be empowered to appoint, with the approval of the Presidents of the Royal Colleges of Physicians and Surgeons, if they should consent to give the matter their consideration, a medical staff to serve for one year, at the end of which time a full report could be laid before the governors, who would then resume their position under the present constitution, or such revision of it as they might agree to.

23. Lastly, we observe that there was a deficit of £777 4s. 8d. on the accounts of 1904, which, with various outstanding debts, shows a deficit of over £1,000 at the present time. We do not see how the arrangement we suggest can be carried out unless the King's Fund is prepared to provide a sum not exceeding £1,000 for the current necessities of the proposed new Board, who would possibly even then have to report to the governors before the expiration of the full year.

Frederick M. Fry.

April 10th, 1905. J. Danvers Power.

At a meeting of the Executive Committee of King Edward's Hospital Fund held on April 11th, 1905, the above report was approved and adopted, and copies were directed to be sent to the officials and governors of the Queen's Jubilee Hospital.

